# Ability to access community-based needle-syringe programs and injecting behaviors among drug users: a cross-sectional study in Hunan Province, China

**DOI:** 10.1186/1477-7517-10-8

**Published:** 2013-05-07

**Authors:** Lei Zhang, Xi Chen, Jun Zheng, Junshi Zhao, Jun Jing, Jun Zhang, Eric PF Chow, David P Wilson

**Affiliations:** 1 The Kirby Institute, The University of New South Wales, Sydney, NSW, Australia; 2 Division of HIV/AIDS and STI control, Hunan Provincial Centers for Disease Control and Prevention, Hunan, China; 3 Comprehensive AIDS Research Center, Tsinghua University, Beijing, China

## Abstract

**Background:**

Needle-syringe exchange programs (NSPs) have been substantially rolled-out in China since 2002. Limited studies reported effectiveness of NSPs in a Chinese setting. This study aimed to assess the association between accessibility to NSPs and drug-use risk behaviors of IDUs by investigating primary (self-reported) data of IDUs recruited from NSP sites, community settings and mandatory detoxification centers (MDCs) in Hunan province, China.

**Methods:**

A cross-sectional survey was conducted in Hunan province in 2010. IDU recruits participated in a face-to-face interview to provide information related to their ability to access NSPs, demographic characteristics, and injecting behaviors in the past 30 days.

**Results:**

Of the total 402 participants, 35%, 14% and 51% participants indicated low, medium and high ability to access NSPs in the past 30 days, respectively. A significantly higher proportion of IDUs (77.3%) from the high-access group reported ≤2 injecting episodes per day compared with medium- (46.3%) and low-access (58.8%) groups. Only 29.0% of high-access IDUs re-used syringes before disposal in the past 30 days, significantly lower than those in the medium- (43.1%) and low-access (41.3%) groups. Reported levels of needle/syringe sharing decreased significantly as the ability to access NSPs increased (16.3%, 12.7% and 2.5% in the low, medium and high access groups, respectively). Ninety percent of IDUs recruited from MDCs had low ability to access NSPs.

**Conclusions:**

Increased NSP accessibility is associated with decreased levels of injecting frequency, repetitive use and sharing of injecting equipment among Chinese IDUs. Mandatory detention of IDUs remains as a major barrier for IDUs to access NSPs in China.

## Introduction

HIV epidemics usually first emerge in China among people who inject drugs. HIV prevalence among injecting drug users (IDUs) in southwest and northwest China has stabilized at ~20% in the past decade [1-4], with infection spreading across to in other at-risk populations and the general population [5,6]. By 2009, 32.2% of the estimated 740,000 people living with HIV in China [7] were infected through sharing of injecting equipment [1]. Needle-syringe exchange programs (NSPs) have been progressively rolled-out in major Chinese cities since 2002 [8]. By 2010, a total of 1023 NSPs sites have been established across 453 Chinese counties [9]. Access by IDUs to NSPs is largely associated with awareness of the programs, extent of police presence and risk of incarceration [10]. Illicit drug use is punishable by law in China. Upon arrest, IDUs are often directed to methadone maintenance therapy (MMT) clinics under supervision of the police. If relapse occurs during treatment, they are transferred to mandatory detoxification centers (MDCs) or labor camps for a period of between one and three years for compulsory drug rehabilitation [11-13]. In these institutions, access to harm reduction services is essentially non-existent. Currently, institution-based interventions, including mandatory detoxification, voluntary rehabilitation and MMT, cover only 15% of the total drug users population [14], indicating a large gap for the scale-up of community-based interventions, such as NSPs.

NSPs have been shown to be a safe and effective means to reduce syringe sharing and subsequent HIV transmission among IDUs in various settings [15-20]. Although NSPs have been implemented for almost a decade in China, only limited studies evaluate the effectiveness of NSPs in alleviating risk behaviors among Chinese IDUs and all have been conducted in high-HIV transmission provinces (Sichuan [21], Guangxi [21,22] and Guangdong [22,23]). Based on a cross-sectional study design, this study further investigates (1) the association between the ability to access NSPs and injecting behaviors in a low-HIV transmission setting; and (2) the impact of mandatory detention on IDUs’ ability to access NSPs.

## Methods

### Study site, design and population

Hunan province is located in South China, bordered by Guangdong and Guangxi provinces to the south and Guizhou province to the west. All three adjacent provinces are traditional drug-trafficking provinces with exceptionally high HIV prevalence levels among IDUs [1]. Hunan’s location has led to its role of channeling illicit drugs to other Chinese provinces [24]. A cross-sectional survey was conducted in three cities (Hengyang, Yiyang and Huaihua) of Hunan province from September to October 2010. Study participants were recruited from the community, NSP sites and MDCs. Community IDUs were recruited by outreach and peer-referral, whereas IDUs from NSP sites and MDC were recruited at venues. In MDCs, only IDUs admitted recently (<3 months) were recruited to recall their injecting behaviors 30 days before their incarceration. Recent MDC entrants were particularly chosen to reduce recall bias.

Upon informed consent, participants completed a 30-minute questionnaire through face-to-face interviews; no identifying personal information was collected. The standardized questionnaire included questions related to demographic characteristics and injecting behaviors in the last 30 days (Table 1). In particular, the participants’ ability to access NSPs was measured as the proportion of needle/syringes obtained from NSPs in the past three months. For incarcerated drug users, this refers to their ability to access before incarceration.

**Table 1:** Demographic Characteristics, NSP accessibility and diseases prevalence levels among recruited IDUs

**Characteristics**	**N**	**Percent (%)**
Venue of recruitment (n = 402)		
*Needle and syringe program*	153	38.1
*Mandatory Detoxification Centers*	78	19.4
*Community*	171	42.5
Gender (n = 402)		
*Male*	333	82.8
*Female*	69	17.2
Age (n = 402)		
<20	4	1
20-29	98	24.4
30-39	180	44.8
40-49	116	28.9
≥50	4	1
Average (±SD)	34.57 ± 7.05	
Ethnic (n = 400)		
*Han*	394	98.5
*Non-Han*	6	1.5
Education (n = 402)		
*Primary or below*	86	21.4
*Junior high school*	252	62.7
*Secondary high school*	54	13.4
*College or above*	10	2.5
Marital Status (n = 402)		
*Single*	166	41.3
*Married and cohabited*	139	34.6
*Divorced and widowed*	97	24.1
Currently employed (n = 395)		
*Yes*	30	7.5
*No*	365	92.5
Respondents' accessibility to NSP (n = 402)		
*High (>70%)*	142	35.3
*Medium (40-70%)*	55	13.7
*Low (<40%)*	205	51.0

### Statistical analysis

Questionnaire data were double-entered and checked in EpiData (v3.0). For analyses, study participants were stratified into groups according to (1) their ability to access NSPs (i.e. low-access [<40%], medium-access [40-70%] and high-access groups [>70%]) and (2) their venue of recruitment (Table 2). Descriptive statistics were calculated in the Statistical Package for the Social Sciences (v19) for Windows. Chi-square tests were used to assess differences between groups.

**Table 2:** Injecting and sharing behaviors among 402 recruited IDUs

	**Overall**	**Ability to access to NSP syringes**^**‡**^			**χ**^**2**^**-test**	**Venue stratification**			**χ**^**2**^**-test**
		**<40%**	**40-70%**	**>70%**	**(χ**^**2**^**, *p*-value)**	**MDC**	**Community**	**NSP**	**(χ**^**2**^**, *p *value)**
	**N (%)**	**N (%)**	**N (%)**	**N (%)**		**N (%)**	**N (%)**	**N (%)**	
Used drugs in the last 30 days (n = 402)*									
*Yes*	398 (99.0)	139 (97.9)	55 (100)	204 (99.5)		74 (94.9)	171 (100)	153 (100)	
*No*	4 (1.0)	3 (2.1)	0 (0)	1 (0.5)	2.89, p = 0.236	4 (5.1)	0 (0)	0 (0)	16.782, p < 0.001†
**Frequency of injecting drugs in the last 30 days (n = 392)**									
*<1 times / day*	41 (10.5)	12 (8.8)	4 (7.4)	25 (12.4)		7 (9.6)	19 (11.1)	15 (10.1)	
*1-2 times / day*	220 (56.1)	68 (50.0)	21 (38.9)	131 (64.9)		37 (50.7)	109 (63.7)	74 (50.0)	
*3-5 times / day*	115 (29.3)	46 (33.8)	28 (51.9)	41 (20.3)		22 (30.1)	39 (22.8)	54 (36.5)	
*>6 times / day*	16 (4.1)	10 (7.4)	1 (1.9)	5 (2.5)	29.439, p < 0.001†	7 (9.6)	4 (2.3)	5 (3.4)	15.236, p = 0.019†
**Times of repeated use per needle/syringe before disposal in the last 30 days (n = 389)**									
*0*	4 (1.0)	0 (0)	3 (5.9)	1 (0.5)		0 (0)	0 (0)	4 (2.7)	
*1*	248 (63.8)	81 (58.7)	26 (51.0)	141 (70.5)		49 (66.2)	111 (65.7)	88 (60.3)	
*2-5*	131 (33.7)	54 (39.1)	22 (43.1)	55 (27.5)		21 (28.4)	57 (33.7)	53 (36.3)	
*6-9*	1 (0.3)	1 (0.7)	0 (0)	0 (0)		1 (1.4)	0 (0)	0 (0)	
*≥10*	5 (1.3)	2 (1.4)	0 (0)	3 (1.5)	6.84, p = 0.033†	3 (4.1)	1 (0.6)	1 (0.7)	0.151, p = 0.927
*Average ± SD*	1.62 ± 1.32	1.80 ± 1.45	1.55 ± 0.86	1.52 ± 1.31		1.93 ± 2.00	1.54 ± 1.05	1.56 ± 1.13	
**Shared syringes in the last 30 days (n = 400)**									
Yes	35 (8.8)	23 (16.3)	7 (12.7)	5 (2.5)		15 (19.5)	4 (2.3)	16 (10.5)	
No	365 (91.2)	118 (83.7)	48 (87.3)	199 (97.5)	21.326, p < 0.001†	62 (80.5)	167 (97.7)	136 (89.5)	20.507, p < 0.001†
**Number of times shared syringes in the last 30 days (n = 29)**									
*1*	10 (34.5)	9 (45.0)	1 (16.7)	0 (0)		8 (66.7)	0 (0)	2 (15.4)	
*2-5*	16 (55.2)	9 (45.0)	5 (83.3)	2 (66.7)		4 (33.3)	3 (75.0)	9 (69.2)	
*≥6*	3 (10.3)	2 (10.0)	0 (0)	1 (33.3)	4.83, p = 0.090	0 (0)	1 (25.0)	2 (15.4)	5.780, p = 0.056
*Average ± SD*	4.28 ± 6.85	4.15 ± 7.37	2.17 ± 0.98	9.33 ± 9.29		1.55 ± 0.93	6.75 ± 8.85	5.92 ± 8.78	
**Number of sharing partners in the last 30 days (n = 31)**									
*1*	10 (31.3)	5 (22.7)	5 (71.4)	0 (0)		4 (28.6)	1 (25.0)	5 (35.7)	
*2-5*	20 (62.5)	15 (68.3)	2 (28.6)	3 (100)		9 (64.3)	3 (75.0)	8 (57.1)	
*6-9*	1 (3.1)	1 (4.5)	0 (0)	0 (0)		0 (0)	0 (0)	1 (7.1)	
*≥10*	1 (3.1)	1 (4.5)	0 (0)	0 (0)	6.73, p = 0.035†	1 (7.1%)	0 (0)	0 (0)	0.594, p = 0.743
*Average ± SD*	2.53 ± 1.98	2.82 ± 2.26	1.43 ± 0.79	3.00 ± 0.0		2.64 ± 2.50	2.25 ± 0.96	2.85 ± 1.99	
**Maximum number of group sharing partners in the last 30 days (n = 32)**									
*1*	2 (6.3)	1 (4.5)	1 (14.3)	0 (0)		1 (7.1)	0 (0)	1 (7.1)	
*2-5*	28 (87.5)	20 (90.9)	5 (71.4)	3 (100)		13 (92.9)	4 (100)	11 (78.6)	
*6-9*	2 (6.3)	1 (4.5)	1 (14.3)	0 (0)	3.19, p = 0.203	0 (0)	0 (0)	2 (14.3)	0.867, p = 0.648
*Average ± SD*	2.94 ± 1.68	2.86 ± 1.42	3.00 ± 2.71	3.33 ± 0.58		2.50 ± 0.76	2.50 ± 0.58	3.50 ± 2.35	

‡ Ability to access to NSP syringes is defined as the proportion of syringes obtained from NSPs during the past 3 months at the time of survey.

† p-value is significantly (<0.05) by Chi-2 test.

* Number in bracket refers to the number of participants who answered the particular question.

## Ethical considerations

Ethics approval was obtained from the Institutional Review Board of the Tsinghua University (Project Code: 0020120508). The survey was collected confidentially and anonymous with no names and personal information obtained. Verbal and written consent procedures were provided to all participants before the survey, and they had the right to withdraw from the study at any time without penalty.

## Results

### Characteristics of study participants

A total of 402 IDUs (community 42.5%; NSP sites 38.1%; MDC 19.4%) participated in this study. Of all participants, 333 were male and 69 were female. Ages ranged between 17 to 56 years (mean 34.6 ± 7.1 years). The majority of respondents was ethnically Han (98.5%), had finished junior high school (84.1%), was currently unemployed (92.5%) and was single/divorced (65.4%) (Table 1). About 35%, 14% and 51% of participants reported low, medium and high ability to access NSPs in the past three months. Ninety percent (90%) of IDUs recruited from MDCs had low ability to access NSPs, much higher than recruits from community (24.6%) and NSP sites (19.0%). In contrast, 62.0% and 61.4% of community and NSP recruits obtained >70% of their syringes from NSPs, whereas only 6.3% of recent MDC entrants obtained this level of their syringes from NSPs.

### Injection risk behaviors

Almost all (99%) IDUs injected and consumed heroin exclusively (97.0%) in the past 30 days. A significantly higher proportion of IDUs in the high-access group (77.3%) reported no more than two injecting episodes per day compared with low (58.8%) and medium-access groups (46.3%) (χ^2^ = 29.44, p < 0.001). When stratified by recruitment venues, the proportion (74.8%) of IDUs reporting no more than two injecting episodes per day is significantly higher among community IDUs than those from NSP sites (60.1%) and MDCs (60.3%) (χ^2^ = 15.24, p = 0.019). Only 29.0% of high-access IDUs reported having re-used syringes before disposal in the past 30 days, significantly lower than the medium and low-access groups (43.1% and 41.3% respectively) (χ^2^ = 6.84, p = 0.033). Pattern of repeated use of syringes did not vary substantially across recruitment venues (Table 2).

Rates of needle/syringe sharing decreased substantially as ability to access NSPs increased (16.3%, 12.7% and 2.5% in the low-, medium- and high-access groups respectively). Respondents from MDCs had the highest syringe sharing rate (19.5%) 30 days prior to their incarceration in comparison with recruits from community (2.3%) and NSP sites (10.5%) (χ^2^ = 20.51, p < 0.001). Among those who shared syringes, an IDU shared on an average of 4.28 ± 6.85 occasions with 2.53 ± 1.98 sharing partners. The average sharing group size is approximately three (Table 2).

## Discussion

Consistent with previous findings in both international [25-30] and Chinese settings [21-23], our findings indicate that provision of clean needle/syringes does not increase the injecting frequency among IDUs, and furthermore that increased availability of clean needles/syringes may substantially contribute to the reduction in repeated use of dull syringes and sharing activities among Chinese IDUs. Liu et.al., in 2007, demonstrate that the rate of syringe sharing in the past 30 days among IDUs who regularly attend NSPs (15%) is two to three times lower than non-attendees (32-44%) in Guangxi and Sichuan provinces [21]. Wu et.al., through a 12-month cohort study, show that the sharing rate dropped from 68% to 35% in the NSP intervention community in South China. Although, in comparison, IDUs in Hunan have a much lower sharing rate in general (low-access group: 16.3%), an increased ability to access NSPs is associated with substantial reductions in sharing behaviors (high-access group: 2.5%).

IDU recruits from MDCs exhibit significantly higher sharing rates, indicating a higher level of risk behaviors. Lower ability to access NSPs prior to their incarceration also suggests that the participation in MMT and likely police supervision may have prevented IDUs from accessing NSPs. In contrast, approximately two-thirds of the community and NSP recruits obtained more than 80% of their syringes from NSPs, suggesting NSPs are generally available and accessible to the majority of IDUs outside of the detention settings. In addition, confinement in MDCs does not reduce drug use behaviors [31-33] and as high as 95% of IDUs relapse for drug addiction within one year of release [34]. Detainees also have limited access to health information and education that are often associated with NSPs. Together with our findings, this indicates that mandatory detoxification may be a major obstacle for harm reduction programs and improving risk behaviors for Chinese IDUs.

The first major limitation of this study is its cross-sectional design. This does not enable us to directly investigate the causal relationship between NSP accessibility and its direct impact on drug use behaviors, although it provides an association analysis between the two. Second, the ability to access NSPs is defined as the self-reported percentage of acquired NSPs syringes in the past three months, which may be subjected to self-recall bias. Third, although the study has been carefully designed to obtain information of injecting behaviors of recent MDC entrants prior to their admittance, the information may underestimate their actual risk behaviors due to prior harm reduction programs (e.g. MMT) [35,36] and the temporary abstinence of injecting behaviors during the transferring process to MDCs by the police.

Our study has clearly demonstrated behavioral improvements when the ability to access NSPs increases in China. In addition, NSPs have been shown to be highly cost-effective in both international [37-40] and Chinese settings [41]. Full scale roll-out of NSPs should be implemented as a major component of harm reduction strategies nationwide. However, continued law enforcement and mandatory detoxification remain as major barriers to the necessary program scale-up and may even counteract the benefits of NSPs [11,42]. Ongoing police crackdowns, arrests and confinement substantially discourage IDUs from contacting peer health educators and accessing NSP sites. In comparison, in places where police are supportive of NSPs, coverage of the programs quickly increased and risk behaviors decreased [43]. Without effective cooperation between legislation, law enforcement and health policy sectors, NSPs are unlikely to reach a sufficient proportion of IDUs to make a significant impact on China’s HIV epidemic.

## Competing interests

The authors declare that they have no competing interests.

## Authors’ contributions

EPFC and LZ performed data analyses and wrote the Method and Result sections of the manuscript. XC, JZ1, JSZ conducted the field study and data collection. JZ2 and JJ participated in study design. LZ and XC wrote the manuscript. DPW assisted with data analyses and was responsible for the supervision of the project. All authors read and approved the final version of the manuscript.
